# Type and duration of water stress influence host selection and colonization by exotic ambrosia beetles (Coleoptera: Curculionidae)

**DOI:** 10.3389/finsc.2023.1219951

**Published:** 2023-07-07

**Authors:** Christopher M. Ranger, Madhav Parajuli, Sean Gresham, Jenny Barnett, Sara Villani, James Walgenbach, Fulya Baysal-Gurel, James S. Owen, Michael E. Reding

**Affiliations:** ^1^Application Technology Research Unit, United States Department of Agriculture (USDA)-Agricultural Research Service, Wooster, OH, United States; ^2^Department of Entomology, The Ohio State University, Wooster, OH, United States; ^3^Department of Agricultural and Environmental Sciences, Otis L. Floyd Nursery Research Center, Tennessee State University, McMinnville, TN, United States; ^4^Department of Entomology and Plant Pathology, Mountain Horticultural Crops Research and Education Center, North Carolina State University, Mills River, NC, United States

**Keywords:** flood stress, drought stress, secondary insects, wood-boring beetles, Scolytinae, Xyleborini, *Xylosandrus germanus*, *Anisandrus maiche*

## Abstract

Fungus-farming ambrosia beetles in the tribe Xyleborini tunnel into plants and trees to establish chambers for cultivating their nutritional fungal mutualists and rearing offspring. Some xyleborine ambrosia beetles preferentially infest and perform better in living but weakened trees. Flood stress predisposes horticultural tree crops to infestation, but the impact of drought stress has not been well studied. Our objectives were to compare the effects of flood stress *vs.* drought stress on host selection and colonization by xyleborine ambrosia beetles and to assess the duration of flooding. Container-grown *Cornus florida* L. trees were flood stressed using a pot-in-pot system to submerge the roots in water while drought-stressed conditions were imposed by withholding irrigation and precipitation. When experimental trees were held under field conditions for 14 days, 7.5 × more ambrosia beetles landed on stems of the flood-stressed than on the drought-stressed trees. During two additional experiments over 14 and 22 days, ambrosia beetles tunneled into the flood-stressed trees but not the drought-stressed or standard irrigation trees. By simultaneously deploying trees that were flood stressed for varying lengths of time, it was found that more tunnel entrances, and xyleborine adults and offspring were recovered from trees that were flooded for 1–16 days and 7–22 days than from trees that were flooded for 14–29 days and 28–43 days. These results indicate that acute and severe drought stress does not predispose *C. florida* to infestation, but flood stress and the duration of flooding influence ambrosia beetle host selection and colonization. Understanding the role of host quality on ambrosia beetle preference behavior will assist with predicting the risk of infestation of these opportunistic insects in horticultural tree crops.

## Introduction

Ambrosia beetles (Curculionidae: Scolytinae) in the tribe Xyleborini are wood-boring insects that can negatively impact horticultural trees growing in ornamental nurseries and orchards (1, 2). Infestations can affect the aesthetic quality of trees and lead to branch dieback and tree death, particularly in saplings and small trees (3–5). The exotic species *Xylosandrus crassiusculus* (Motschulsky) and *Xylosandrus germanus* (Blandford), both of which are native to East and South Asia, are among the most problematic ambrosia beetles in nurseries and orchards in North America (3–5). *X. crassiusculus* was first detected in South Carolina in 1974 and is established in 31 states along with the Canadian province of Ontario (6–8). *X. germanus* was first detected in New York in 1932 and is established in 34 states and the Canadian provinces of British Columbia, Nova Scotia, Ontario, and Québec (7, 8). *Anisandrus maiche* Stark is native to East Asia and was first detected in North America in 2009; it has since spread to eight states within the Midwestern and Eastern USA (9, 10). As an emerging insect pest, *A. maiche* has become a prominent exotic species recovered from experimentally stressed trees in Ohio (11) and baited traps in Indiana (12).

As fungus-farming insects, xyleborine ambrosia beetles tunnel into the stems and branches of trees where they inoculate host tissues with their nutritional fungal mutualist on which the larvae and adults must feed (1). *Ambrosiella cleistominuta* Mayers is the fungal mutualist of *A. maiche* (13), *Ambrosiella roeperi* Harrington and McNew is the fungal mutualist of *X. crassiusculus* (14), and *Ambrosiella grosmanniae* Mayers, McNew, and Harrington is the mutualist of *X. germanus* (15). Both *X. crassiusculus* and *X. germanus* select from a broad range of 100–200 host tree species when attempting to establish their fungal mutualists and rear offspring (2). *A. maiche* is associated with a broad range of host tree species (10).

Like other wood-boring insects (16), *X. crassiusculus* and *X. germanus* have been demonstrated to preferentially attack and perform better on experimentally stressed than on “apparently healthy” host trees (2). Under free-choice conditions, *X. crassiusculus* and *X. germanus* preferentially tunneled into flood- or freeze-stressed trees, but rarely tunneled into untreated control trees (2, 5, 17, 18). Under no-choice conditions, *X. crassiusculus* and *X. germanus* established fungal gardens and produced offspring in the stems of flood-stressed *Cornus florida* L. but not non-flooded trees (19). The duration of flood stress also influences ambrosia beetle host selection, whereby the number of ambrosia beetle tunnels and offspring production tended to increase as the flood duration of *Malus* × *domestica* Borkh., *C. florida*, and *Cercis canadensis* L. increased (18).

Drought stress predisposes individual trees and entire forests to outbreaks of bark beetles (20, 21), but few studies have assessed the influence of drought stress on host selection and colonization by xyleborine ambrosia beetles. *Acacia koa* Gray, *Croton reflexifolius* Kunth., and *Coffea* spp. plants infested by *Xylosandrus compactus* Eichhoff were anecdotally reported as being predisposed, in part, to drought stress (22, 23). In contrast, *C. florida* trees maintained at 90% and 70% media moisture were attacked by ambrosia beetles, but trees maintained at 50% and 30% media moisture were not preferred (24). Since container- and field-grown trees in horticultural cropping systems can be exposed to water-stress conditions, it is important to understand the role of drought and flood stress on tree attractiveness and suitability to ambrosia beetles. Notably, flood stress and drought stress induce the production and emission of host-derived ethanol, which represents a key attractant for ambrosia beetles (19, 25–27).

Based on the aforementioned studies, we hypothesized that ambrosia beetle host selection is influenced by the type of water stress (i.e., flooding *vs*. drought) and the duration of flood stress. The following objectives were addressed as part of our current study: (i) compare the effects of flood stress *vs.* drought stress on host selection and colonization by *A. maiche*, *X. crassiusculus*, and *X. germanus* and (ii) assess if the duration of flood stress influences ambrosia beetle host selection and colonization.

## Materials and methods

### Attraction to water-stressed trees

Attraction of ambrosia beetles was compared using 2- to 3-year-old flowering dogwood, *C. florida* L., about 0.9 m in height, and growing in 19-L pots that were subjected to flood stress, drought stress, or standard irrigation treatments. *C. florida* was used due to its intolerance to flood stress and suitability as a host for ambrosia beetles (19, 26). On 28 June 2016, potted *C. florida* trees were arranged in six randomized complete blocks consisting of one tree per block receiving the flood stress, drought stress, or standard irrigation treatment. There were 3 meters between adjacent trees within a block and 6 meters between adjacent blocks. Trees were deployed within a deciduous woodlot in Wayne Co., Ohio (40°45′40.85′N, 81°51′14.71′W).

**Figure 1:**
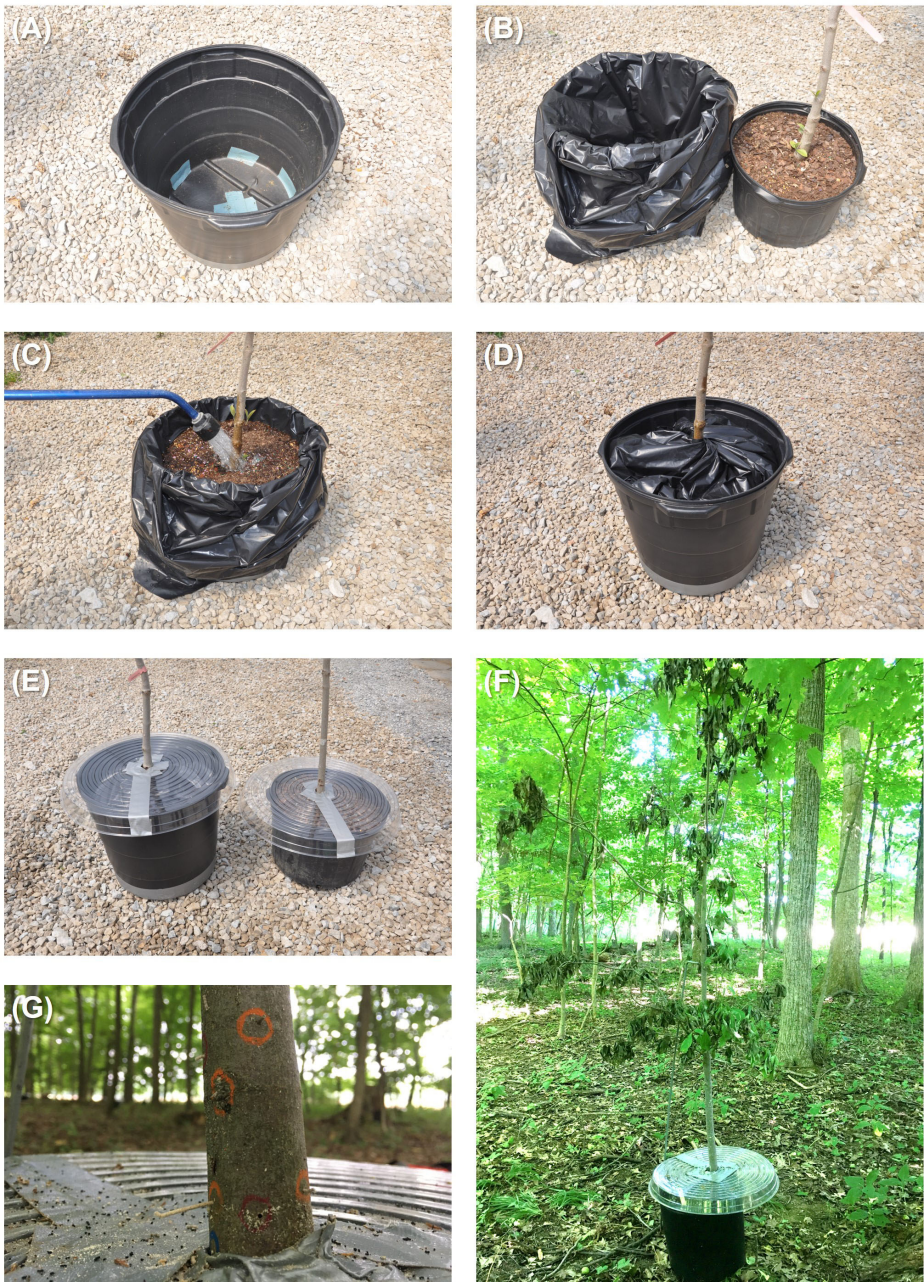
Flood-stress conditions were imposed using a pot-in-pot system whereby **(A)** the drainage holes on a 26-L pot were first covered with duct tape and then the **(B)** pot was lined with a plastic bag. **(C)** A 19-L pot containing a *Cornus florida* tree was placed inside the 26-L pot lined with a plastic bag and irrigated until standing water covered the soil surface. **(D)** The excess plastic bag was then twisted and tucked in between the inner and outer pots to prevent ambrosia beetles from landing in the standing water. **(E)** Drought stress was imposed using rain deflectors to cover pots containing *C. florida* trees. Rain deflectors were prepared using clear houseplant plastic drip saucers. **(F)** Drought-stressed *C. florida* tree deployed within a woodlot. **(G)** Ambrosia beetle sawdust “noodles” and tunnel entrances created in a flood-stressed *C. florida* tree (circled with wax pencils).

Trees within each block were randomly subjected to flood stress, drought stress, or standard irrigation. Following Ranger et al. (26), flood-stress conditions were imposed using a pot-in-pot system whereby a 19-L pot containing a *C. florida* tree was placed inside a 26-L pot lined with a plastic bag and irrigated until standing water covered the soil surface (**Figure 1**). Flood-stress conditions were maintained throughout the duration of the experiment. Drought stress was initiated by ceasing irrigation and using rain deflectors to cover the pots (**Figure 1**). Rain deflectors were prepared using clear houseplant plastic drip saucers (45.7 cm in diameter; Hawthorne Gardening Co., Vancouver, WA). Scissors were used to cut along the radius to the center of the saucer, after which a 3-cm-diameter circle was cut in the center of the saucer. The saucer was then inverted and a *C. florida* stem was arranged to extend through the opening in the center of the saucer. The inverted saucer was laid on top of the pot, and duct tape was then used to seal the radius and the junction around the center of the saucer and the stem. Rain deflectors were also secured over pots containing the flood-stressed and standard irrigation trees to avoid any bias associated with reflectance of the clear plastic saucers. Trees assigned to the standard irrigation treatment received 1L of water every 3–4 days.

Tanglefoot^®^ (The Tanglefoot Company, Grand Rapids, Michigan) was next applied using a paintbrush in a continuous spiral pattern (2.54 cm in width) up to 40 cm from the base on stems of all trees, following Ranger et al. (26). A corresponding spiral pattern of stem tissue without Tanglefoot (2.54 cm in width) was retained to facilitate the natural emission of attractive semiochemicals from the bark surface. The incidence of landing on stems of the experimental trees was used as an indicator of ambrosia beetle attraction. Tanglefoot was applied on the same day that the trees were deployed under field conditions and the flood-stress and drought-stress conditions were initiated. Ambrosia beetles were carefully removed using forceps from the Tanglefoot every 1 – 4 days throughout the duration of the experiment and stored in vials with 70% ethanol for subsequent identification of species. Trees were deployed under field conditions on 28 June 2016 and maintained until 12 July 2016 for a total of 14 days.

Ethical review and approval was not required for this study on ambrosia beetles in accordance with local legislation and institutional requirements. No protected species were sampled during the course of the experiments.

### Host selection and colonization of water-stressed trees

Ambrosia beetle selection and colonization of *C. florida* trees subjected to flood stress, drought stress, and a standard irrigation schedule were assessed during two separate experiments under field conditions. As described below, the concentration of ethanol within stems of flood-stressed, drought-stressed, and standard irrigation trees was also analyzed as part of the first experiment. The relative volumetric water content of the growing media subjected to drought-stress and standard irrigation conditions was measured during the second experiment as described below.

#### Experiment 1

*C. florida* trees were arranged on 26 July 2016 in six randomized complete blocks within a deciduous woodlot (40°45′40.85′N, 81°51′14.71′W) with one tree per block receiving the flood-stress, drought-stress, or standard irrigation treatment. Trees were spaced 3 meters apart within a block and 6 meters between adjacent blocks. Flood-stress and drought-stress treatments were initiated as previously described on the day of field deployment. Trees were thoroughly examined for ambrosia beetle tunnel entrances every 1–3 days over a total duration of 14 days. On the last day of the experiment (8 August 2016), tissue samples were taken from the experimental trees to analyze for ethanol, as described in detail by Ranger et al. (26). Briefly, four tissue core samples (1 mm in depth, 5 mm in diameter) were collected per stem using an Osborne arch punch (C.S. Osborne & Co., Harrison, New Jersey). The tissue cores were collected at 10 cm above the soil line and contained the outer bark, phloem, and vascular cambium. Six trees were sampled for the flood-stressed, drought-stressed, and standard irrigation treatments. Tissue core samples were stored at –40°C until analysis.

To analyze for ethanol, the four tissue core samples per tree were transferred to a 2-mL glass vial with a screw top cap and septum, which was then suspended in a water bath at 100°C for 30 min. Vials were removed from the water bath, and a solid phase microextraction (SPME) fiber (carboxen™/polydimethylsiloxane; Sigma-Aldrich, St. Louis, Missouri) was exposed to the headspace volatiles within the vial for 5 min. Fibers were thermally desorbed for 2 min at 250°C in the injection port of an Agilent 7890B GC (Agilent Technologies, Palo Alto, California) with a SPME liner (0.75 mm × 6.35 mm × 78.5 mm, i.d. × o.d. × length; Restek, Bellefonte, Pennsylvania) under splitless mode. A DB-5MS column (0.25 mm × 30 m × 0.25 μm; i.d. × length × film thickness; cross-linked/surface bonded 5% phenyl, 95% methylpolysiloxane; Agilent J&W, Santa Clara, California) was used with a temperature program of 40°C for 2 min followed by a ramp at 15°C/min to 200°C. An Agilent 5977A mass spectrometer was operated in electron impact mode with a scan range of 33–120 amu. External standards of ethanol and a standard concentration curve was used to determine the relative quantities of ethanol associated with the tissue samples.

#### Experiment 2

During the second host selection and colonization experiment, *C. florida* trees were arranged on 5 July 2020 in eight randomized complete blocks within a deciduous woodlot (40°45′40.85′N, 81°51′14.71′W) with one tree per block receiving the flood-stress, drought-stress, or standard irrigation treatment. Trees were spaced 3 meters apart within a block and 6 meters apart between adjacent blocks. Flood-stress and drought-stress treatments were initiated as previously described on the day of field deployment. Trees were thoroughly examined for ambrosia beetle tunnel entrances every 1–3 days for a total duration of 14 days. The relative volumetric water content (i.e., ratio of the volume of water to the unit volume of soil) of the growing media subjected to drought-stress and standard irrigation conditions was also measured under field conditions throughout the second experiment. Specifically, a soil moisture probe was inserted into the media at the four cardinal directions for each pot in which the drought-stressed (n = 8) and standard irrigation (n = 8) trees were growing. Water content was measured from the drought-stressed and standard control trees at 3, 11, and 19 days after they were deployed under field conditions. Water content was not measured in the flood-stressed trees because standing water was maintained throughout the duration of the experiment. The experimental trees were held under field conditions for a total of 22 days. On the final day of the experiment (27 July 2020), the stems were cut at the soil line and transferred to a walk-in cooler maintained at 5°C. Tunnels and galleries within the stems were dissected under laboratory conditions to recover ambrosia beetle foundresses and offspring. Ambrosia beetle specimens were stored in 70% ethanol and identified by species.

### Influence of flooding duration on host selection and colonization

Ambrosia beetle host selection of trees subjected to varying durations of flooding was assessed using *C. florida*. To initiate the experiment, *C. florida* trees were transferred from an exterior USDA-ARS nursery production facility into a greenhouse to impose flood-stress conditions and exclude field-dispersing ambrosia beetles from infesting the trees. Trees were then randomly assigned to be subjected to flood stress for varying durations. Specifically, flooding of the *C. florida* trees was initiated at 28, 14, 7, and 1 day(s) prior to their simultaneous deployment under field conditions. Flood-stress conditions were initiated as previously described. Standing water conditions were maintained until the day of field deployment when the pots were temporarily drained to facilitate moving the trees to a deciduous woodlot in Wayne Co., Ohio (40°47′3.13′N, 81°50′6.21′W).

On the day of field deployment, the trees were arranged in six randomized complete blocks on 16 June 2015 with one tree per block representing a flood duration of 0, 1, 7, 14, or 28 days. A distance of 3 meters was maintained between adjacent trees within a block and 6 meters between adjacent blocks. Flooding of the trees was then immediately reimposed, along with the untreated control *C. florida* trees receiving a standard irrigation regime. Flood stress was maintained while the trees were deployed under field conditions for an additional 15 days, resulting in the total duration of flooding under field conditions being 0, 1–15, 7–22, 14–29, and 28–43 days. Trees were thoroughly examined every 1–3 days, and ambrosia beetle tunnel entrances were counted and circled with a wax pencil. On the last day of field deployment (1 July 2015), the stems were cut at the soil line and transferred to a walk-in cooler maintained at 5°C. Tunnels and galleries within the stems were then dissected using pruning shears to recover the foundress ambrosia beetles. Ambrosia beetle specimens were stored in 70% ethanol and identified by species.

### Statistical analyses

Time-course count data of cumulative tunnel entrances per tree and entrapped ambrosia beetles per tree were first analyzed using repeated measures analysis of variance (ANOVA) (α = 0.05; SAS Institute). When a significant between-subject treatment × time effect was detected (*p <*0.05), the count data associated with specific time points were compared using generalized linear models (SAS Institute). Count data of ambrosia beetles and offspring excavated from infested trees were also analyzed using generalized linear models. Due to non-normality, negative binomial distributions and log link functions were used to fit the models as confirmed by values close to 1.0 for the scaled deviance (G^2^/df) parameter (28). Differences of least squares means were used for pairwise comparisons of treatment effects (α = 0.05).

A repeated measures ANOVA (α = 0.05; SAS Institute) was also used to analyze between-subject and within-subject effects in the relative volumetric water content of growing media associated with drought-stressed and standard irrigation treatments. An unpaired *t*-test (α = 0.05) was then used to compare water content at specific time points between the drought-stressed and standard irrigation treatments. A Pearson correlation coefficient analysis was also used to determine the degree of correlation between the water content of the growing media and days after initiating the drought-stress treatment (SAS Institute).

## Results

### Attraction to water-stressed trees

**Figure 2:**
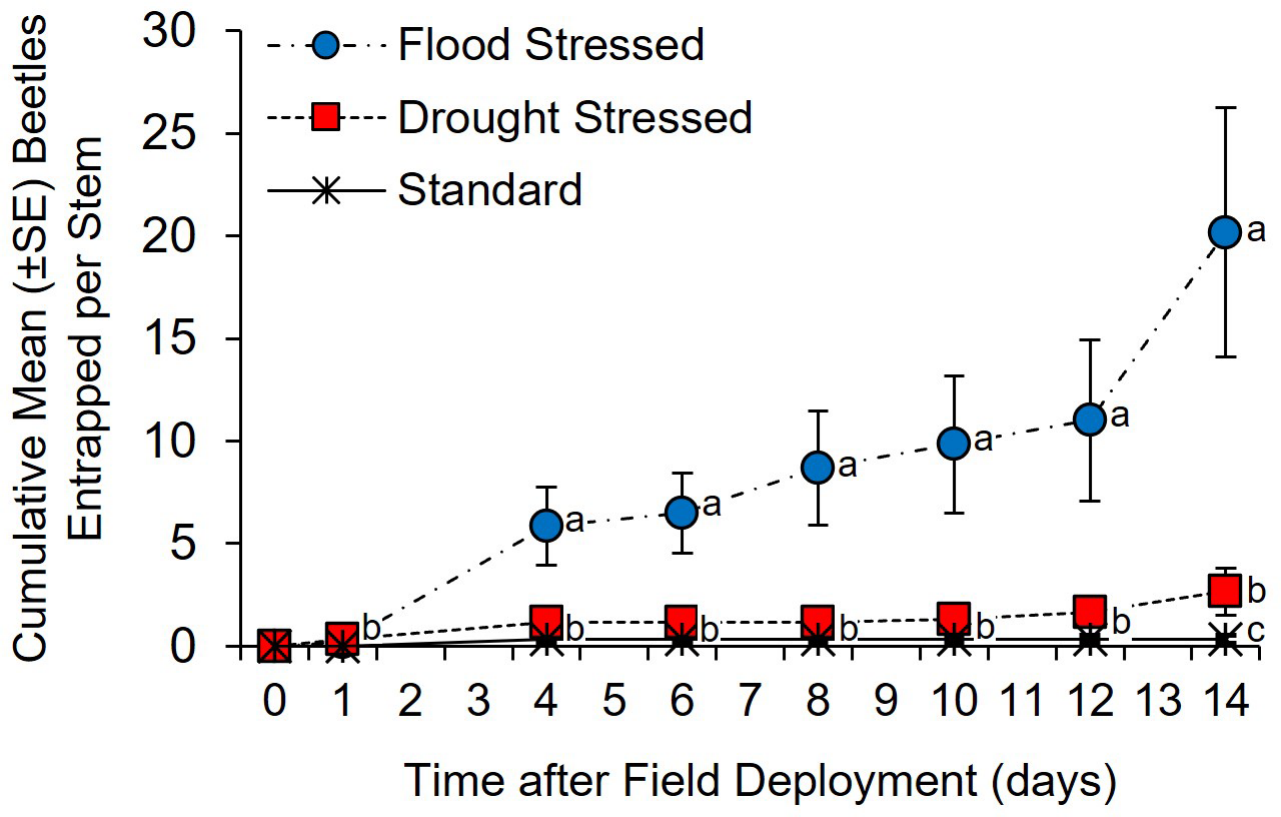
Cumulative *Xylosandrus germanus* attracted to flood-stressed, drought-stressed, and standard irrigation *Cornus florida* trees as measured by the number of beetles entrapped in adhesive Tanglefoot applied to the main stem. Different letters indicate significant differences in the mean number of cumulative *X. germanus* entrapped per tree on specific days using a general linear model and least squares means (α = 0.05; see **Table S1** for statistical output).

*Xylosandrus germanus* was the only species collected from Tanglefoot-coated trees. A significant between-subjects treatment × time effect was detected in the number of beetles entrapped by Tanglefoot on *C. florida* trees subjected to flood-stress, drought-stress, and standard irrigation conditions (**Figure 2**) (*F* = 7.87; df = 2, 15; *p* = 0.0005). No difference in entrapped beetles among treatments was detected at 1 day after field deployment. By day 4, significantly more *X. germanus* were entrapped on stems of the flood-stressed *C. florida* than on drought-stressed and standard irrigation trees (**Table S1**) (χ^2 ^= 13.05; df = 2; *p <*0.0001). Significantly more cumulative *X. germanus* were also entrapped on stems of the flood-stressed *C. florida* at 6, 8, 10, 12, and 14 days after field deployment (**Table S1**). By day 14, significantly more *X. germanus* were also entrapped on stems of the drought-stressed *C. florida* trees than on the standard irrigation trees, but no differences were detected prior to this timepoint (**Figure 2**; **Table S1**).

### Host selection and colonization of water-stressed trees

**Figure 3:**
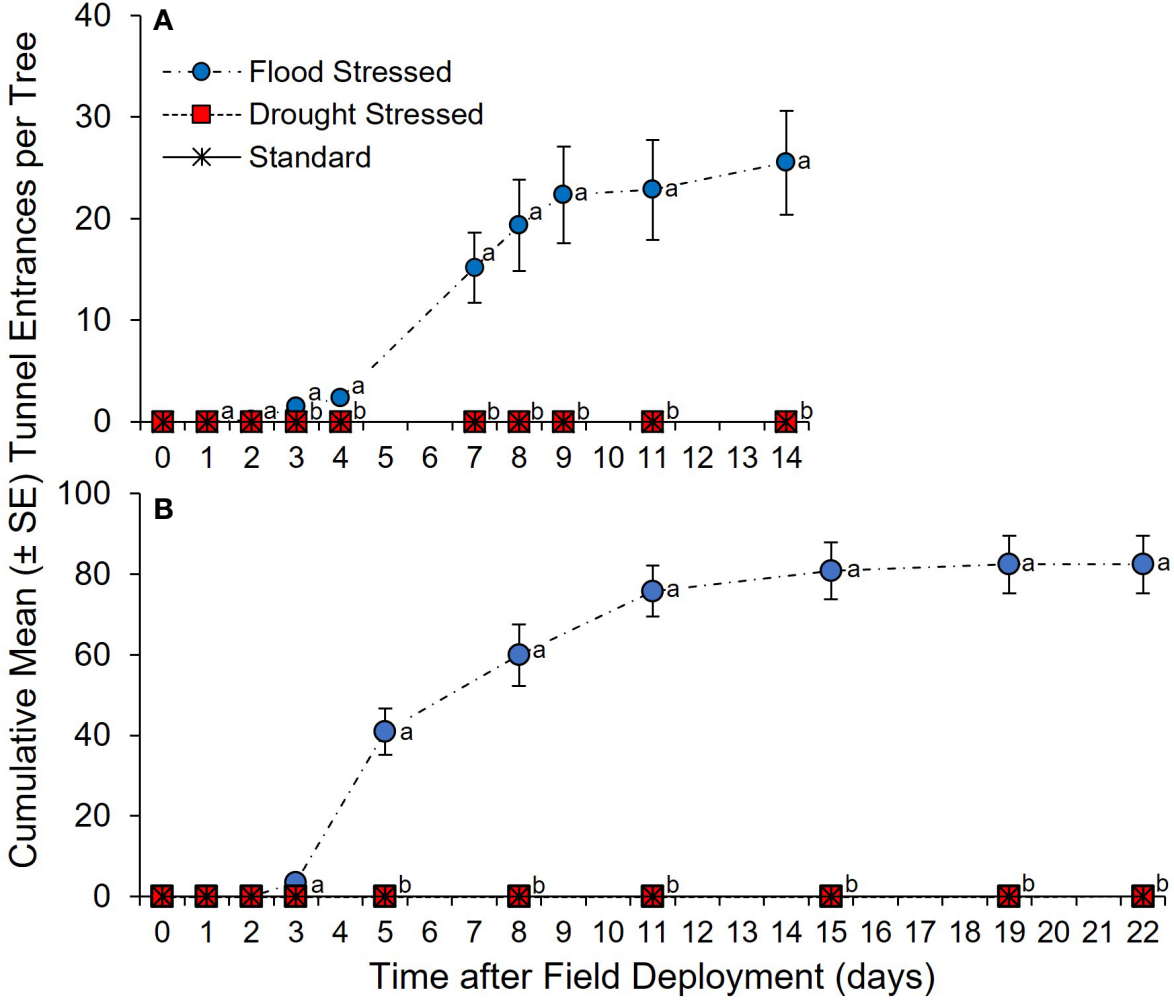
**(A, B)** Cumulative ambrosia beetle tunnel entrances on flood-stressed, drought-stressed, and standard irrigation *C. florida* trees. Trees were deployed on **(A)** 26 July 2016 and **(B)** 5 July 2020. Different letters indicate significant differences in the mean number of cumulative ambrosia beetle tunnel entrances per tree on specific days using a general linear model and least squares means (α = 0.05; see **Tables S2**, **S3** for statistical output).

Two field experiments further evaluated ambrosia beetle host selection of water-stressed *C. florida*. For the first experiment, a significant between-subjects treatment × time effect was detected in the number of tunnel entrances created in flood-stressed, drought-stressed, and standard irrigation trees (*F* = 22.98; df = 2, 15; *p <*0.0001) (**Figure 3A**). No difference in ambrosia beetle tunnel entrances was detected among the flood-stressed, drought-stressed, and standard irrigation trees at 1 and 2 days after field deployment (**Table S2**). In contrast, significantly more cumulative ambrosia beetle tunnel entrances were detected on the flood-stressed *C. florida* trees than on the drought-stressed and standard irrigation trees at 3, 4, 7, 8, 9, 11, and 14 days after field deployment (**Figure 3A**; **Table S1**). No ambrosia beetle tunnel entrances were detected in the drought-stressed or standard irrigation trees. Analysis by SPME-GC-MS detected 3.8 ± 0.8 µg of ethanol per g of stem tissue associated with the flood-stressed trees at 14 days after initiating the stress treatment. Ethanol was not detected in tissue samples from the drought-stressed trees or standard irrigation trees.

For the second experiment, a significant between-subjects treatment × time effect was detected in the number of tunnel entrances created in flood-stressed, drought-stressed, and standard irrigation trees (*F* = 120.90; df = 2, 21; *p <*0.0001) (**Figure 3B**). No difference in ambrosia beetle tunnel entrances was detected among the flood-stressed, drought-stressed, and standard irrigation trees at 1, 2, and 3 days after field deployment (**Table S3**). However, significantly more cumulative ambrosia beetle tunnel entrances were detected on the flood-stressed *C. florida* trees than on the drought-stressed and standard irrigation trees at 5, 8, 11, 15, 19, and 22 days after field deployment (**Figure 3B**; **Table S3**). No ambrosia beetle tunnel entrances were detected in the drought-stressed or standard irrigation trees. Significantly more *X. germanus* and *A. maiche* were recovered from stems of the flood-stressed trees than *X. crassiusculus* (**Table 1**). Dissection of the stems from the flood-stressed trees revealed a total of 182 *X. germanus* (59.3%), 112 A*. maiche* (36.5%), and 13 *X. crassiusculus* (4.2%) (**Table 1**). Significantly more larvae and pupae of *X. germanus* were also recovered from galleries created in stems of the flood-stressed trees than larvae and pupae of *A. maiche* and *X. crassiusculus* (**Table 1**).

**Table 1:** Ambrosia beetles excavated from flood stressed *C. florida* trees (See **Figure 3B**).

Species	Mean (±SE) Specimens per Tree^b^							
	Adults		Eggs		Larvae		Pupae	
*X. germanus*	22.8	± 3.6a	3.9	± 2.1a	101.4	± 12.7a	62.5	± 9.2a
*A. maiche*	14.0	± 2.6a	1.5	± 1.5a	27.9	± 16.5b	19.5	± 6.8b
*X. crassiusculus*	1.6	± 0.4b	0.0	± 0.0a	3.3	± 1.8c	2.3	± 0.9c
Abandoned^a^	13.4	± 2.2	1.1	± 0.7	73.8	± 10.0	48.0	± 12.6
Statistics (χ^2^; *P*)	31.7; <0.0001		5.32; <0.07		19.42; <0.0001		22.32; <0.0001	

a Galleries absent of an adult foundress but containing eggs, larvae, and/or pupae.

b Different letters within a column indicate significant differences among species in the mean number of adults, eggs, larvae, or pupae using generalized linear models and least square means (df = 2 for all comparisons). Data from abandoned galleries were excluded from analyses.

Analysis of the relative volumetric water content of the media associated with *C. florida* trees subjected to drought-stress and standard irrigation treatments detected a significant between-subjects treatment × time effect (*F* = 130.97; df = 1, 14; *p <*0.0001) (**Table 2**). Significantly lower water content was measured from the growing media of the drought-stressed trees than from the standard irrigation trees at 3, 11, and 19 days after initiating the stress treatment (**Table 2**). Water content of the media associated with the drought-stressed trees decreased over time, as indicated by a significant within-subject treatment × time effect (*F* = 30.48; df = 2, 28; *p <*0.0001) and a –0.81 Pearson correlation coefficient (r^2 = ^0.66; *p <*0.0001) (**Table 2**). Significant differences in water content were detected among all three of the sampling dates for the growing media of the drought-stressed trees (**Table 2**). No significant difference was detected in water content among the three sampling dates for the standard irrigation trees.

**Table 2:** Relative volumetric water content (%) of media containing *Cornus florida* subjected to drought stress and a standard irrigation schedule (see **Figure 3B**).

Treatment	Duration after Field Deployment (days)^a^						Statistics (χ^2^; *P*)^a^
	3		11		19		
Drought Stressed	9.6	± 1.4Aa	3.5	± 0.6Ba	1.1	± 0.3Ca	33.56; <0.0001
Standard	20.2	± 1.3Ab	20.9	± 1.3Ab	25.0	± 1.9Ab	4.77; 0.092
Statistics (*t*; *P*)^a^	5.44; <0.0001		11.92; <0.0001		12.20; <0.0001		

a Different uppercase letters indicate significantly different mean volumetric water content within a row by a generalized linear model and least squares means (df = 2). Different lowercase letters indicate significantly different mean volumetric water content within a column by an unpaired t-test (df = 14).

### Influence of flood duration on host selection and colonization

**Figure 4:**
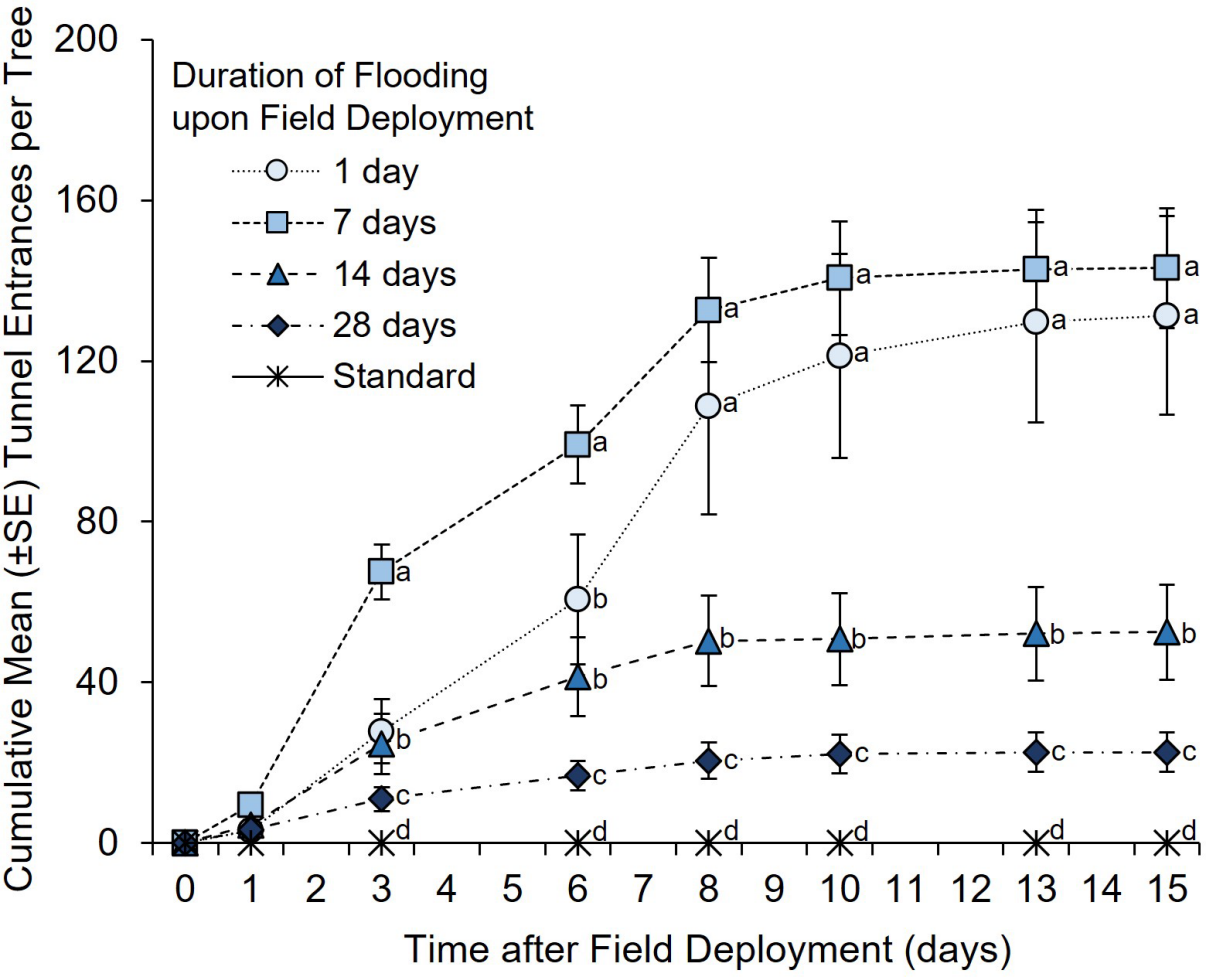
Ambrosia beetle preference for *C. florida* trees subjected to varying durations of flood stress prior to being simultaneously deployed under field conditions. Specifically, trees were flooded under greenhouse conditions and then simultaneously deployed after 0, 1, 7, 14, and 28 days of flooding. Trees were deployed on 16 June 2015 and remained flooded under field conditions for an additional 15 days. Non-flooded control trees received a standard irrigation every 3–4 days. Different letters indicate significant differences in the mean number of cumulative ambrosia beetle tunnel entrances per tree on specific days using a general linear model and least squares means (α = 0.05; see **Table S4** for statistical output).

A significant between-subjects treatment × time effect was detected in the number of tunnel entrances per tree after ambrosia beetles were given free choice among *C. florida* trees that were flood stressed for 0, 1, 7, 14, and 28 days and then simultaneously deployed (*F* = 20.44; df = 4, 25; *p <*0.0001) (**Figure 4**). At 1 day after their simultaneous deployment, significantly more ambrosia beetle tunnel entrances were detected on trees that were deployed after 7 days of flooding than on trees that were deployed after 1 and 28 days of flooding and on the non-flooded controls (χ^2 = ^25.36; df = 4; *p <*0.0001) (**Figure 4**). By 3 and 6 days after their simultaneous field deployment, ambrosia beetle tunnel entrances were higher on trees that were deployed after 7 days of flooding than on trees that were deployed after 0, 1, 14, and 28 days of flooding (**Table S4**; **Figure 4**). By 8, 10, 13, and 15 days after their simultaneous deployment, ambrosia beetle tunnel entrances were higher on trees that were deployed after 1 and 7 days of flooding than on trees deployed after 0, 14, and 28 days of flooding (**Table S4**; **Figure 4**). At the termination of the experiment, the fewest tunnel entrances among the flood-stressed trees were associated with trees that were deployed after 28 days of flooding. No tunnel entrances were recorded from the trees that received a standard irrigation protocol.

Upon dissection of the *C. florida* stems, a total of 1,422 *X. germanus* (72.8%), 524 A*. maiche* (26.8%), five *X. saxesenii* (0.3%), and three *X. crassiusculus* (0.2%) were recovered from the flood-stressed *C. florida* trees. More *X. germanus* and *A. maiche* were recovered from trees deployed after 1 and 7 days of flooding than from trees deployed after 14 and 28 days of flooding (**Table 3**). More *X. germanus* and *A. maiche* were also recovered from trees deployed after 14 days of flooding than from trees deployed after 28 days of flooding (**Table 3**).

**Table 3:** Ambrosia beetles excavated from *C. florida* trees that were subjected to varying durations of flooding and then simultaneously deployed under field conditions; flood stress conditions were then maintained throughout the remainder of the experiment (see **Figure 4**).

		Mean (±SE) per Tree^a^							
Duration of Flooding upon Field Deployment (days)	Duration of Flooding under Field Conditions (days)	*X. germanus*		*A. maiche*		*X. saxesenii*		*X. crassiusculus*	
0	0	0.0	± 0.0d	0.0	± 0.0c	0.0	± 0.0a	0.0	± 0.0a
1	1–16	83.5	± 18.3a	48.8	± 9.6a	0.3	± 0.3a	0.5	± 0.2a
7	7–22	88.0	± 10.6a	36.0	± 18.5a	0.3	± 0.3a	0.0	± 0.0a
14	14–29	42.7	± 9.3b	2.3	± 1.5b	0.2	± 0.2a	0.0	± 0.0a
28	28–43	22.8	± 5.1c	0.2	± 0.2c	0.0	± 0.0a	0.0	± 0.0a
Statistics (χ^2^; *P*)		65.4; <0.0001		43.0; <0.0001		3.06; 0.5		1.88; 0.7	

a Different letters within a column indicate significant differences in the mean number of ambrosia beetles per tree using generalized linear models and least square means.

## Discussion

Warming global temperatures and changes in precipitation patterns are predicted to limit water availability and increase the severity of drought stress (29–31). The growth of horticultural tree crops is negatively affected by water deficits, which also produce an increased risk of secondary outbreaks of insect pests (20, 32, 33). Characterizing the impact of physiological stressors on tree susceptibility to insects is crucial for predicting and managing infestations, especially for wood-boring insects, because they tend to prefer and perform better on stressed hosts (16). Drought-stressed trees with weakened defenses facilitate mass outbreaks of secondary bark beetles within forested ecosystems (34–38). Several studies have demonstrated that flooding predisposes horticultural trees to infestation by ambrosia beetles, but the impact of drought stress on host tree selection and colonization by ambrosia beetles has not been well studied (2).

A previous study demonstrated that *C. florida* trees maintained at 90% and 70% media moisture were subsequently infested by ambrosia beetles, but trees maintained at 50% and 30% media moisture were not selected, and only a single ambrosia beetle tunnel entrance was found on trees maintained at 10% media moisture (24). Our current study further demonstrates that the type and duration of water stress play an important role during host selection and colonization by xyleborine ambrosia beetles. Severe flood stress, but not drought stress, induced attacks and facilitated colonization by ambrosia beetles, particularly *X. germanus* and *A. maiche*. The duration of flood stress also influenced host selection by xyleborine ambrosia beetles, whereby *A. maiche* and *X. germanus* tended to select trees flooded for 1–14 days rather than those flooded for 14–43 days. Thus, not all stressors are equally beneficial to host selection and colonization by xyleborine ambrosia beetles. A broad host range, but narrow preference for a certain type of host quality, could be driven by the life history of ambrosia beetles, including the necessity to select a host substrate conducive to establishing and cultivating their fungal symbionts.

The preference for flood-stressed over drought-stressed *C. florida* trees could be attributed to the emission of long-range volatile kairomones. Based on the landing rates of *X. germanus*, flood-stressed *C. florida* trees were more attractive than drought-stressed trees. Landing rates of *X. germanus* were 7.5 times higher on the flood-stressed trees than on the drought-stressed trees and slightly higher on the drought-stressed trees than on the standard irrigation trees. Attraction to the flood-stressed and drought-stressed *C. florida* is presumably due, in part, to the emission of ethanol, which is a long-range host location cue for ambrosia beetles (2, 39). During a subsequent experiment as part of our current study in which attacks occurred on the flood-stressed *C. florida* but not the drought-stressed trees, ethanol was detected in the stems of the flood-stressed trees but not the drought-stressed or standard irrigation trees. Still, drought stress has the potential to induce the production and emission of ethanol as indicated by previous studies involving conifers. For example, concentrations of ethanol in branches of Douglas fir (*Pseduotsuga menziesii* (Mirb.) Franco) were higher than in untreated trees 3 weeks after breaking the water column (25). Under drought-stress conditions, ethanol concentrations were comparatively higher in needles, sapwood, and phloem of drought-sensitive *P. menziesii* than in drought-tolerant *Pinus ponderosa* Douglas ex C. Lawson (40). Notably, stems of naturally drought-stressed Aleppo pine (*Pinus halepensis* Mill.) under attack by Mediterranean pine shoot beetles (*Tomicus destruens* Wollaston) contained 2 × higher concentrations of ethanol than neighboring attack-free trees (27).

In addition to being a long-range attractant, ethanol also acts as a tunneling cue for *X. germanus* and benefits the growth of their fungal mutualist and host-tree colonization (39, 41, 42). Upon landing on and sampling the host tree, contact cues such as water content might also stimulate ambrosia beetle tunneling and the subsequent establishment of their fungal mutualist(s). Stem water content was not measured as part of our current study, but previous studies have demonstrated that decreased water content in the phloem and sapwood can follow severe drought-stress conditions. For instance, severely water-stressed *P. menziesii* had a lower water content and higher ethanol concentrations in sapwood tissues than in those of the more drought-tolerant *P. ponderosa* (40). Lower water content and higher ethanol concentrations were also detected in the phloem and sapwood of drought-stressed *P. halepensis* attacked by *T. destruens* than in those of adjacent trees that were not attacked (27).

For our current study, severe and acute drought stress was imposed on container-grown *C. florida* by withholding water over 22 days and using modified houseplant plastic drip saucers as rain deflectors. Time-course measurements of the relative volumetric water content of the growing media demonstrated that withholding irrigation and rainwater effectively decreased soil moisture over the course of the study. Specifically, the relative volumetric water content associated with the drought-stress treatment decreased over time to a mean of 1.1% at 19 days after field deployment. In contrast, the mean relative volumetric water content of the standard irrigation trees was 22.0% over the 19-day duration. Relative volumetric water content in the range of 25%–45% is considered field capacity for control plants during drought-stress studies, and a drought stress targeting 5%–10% VWC achieved significant physiological responses in a range horticultural species (43). Wilted and chlorotic leaves of the drought-stressed trees at the termination of the experiment after 22 days represented an additional indicator that drought stress had been imposed. Wilting and damage to photosynthetic processes are a function of severe drought-stress conditions (44, 45).

Our current study relied on passive drying of the media by withholding irrigation, which does not adequately mimic natural soil water deficits (46) but can mimic fast drying of the media within a nursery container production system. Rather than imposing acute and severe drought-stress conditions under which trees rapidly shut down and senesce, future studies could benefit by delivering a minimal amount of water to balance water loss from transpiration and maintain the media at a specific water content (47). For instance, Marchin et al. (43) modified the “Snow and Tingey system” to impose different intensities and durations of water deficit and then measured physiological parameters of plant health, including stomatal conductance, turgor loss (i.e., wilting) point, leaf water potential, and leaf temperatures. The production and emission of ethanol from drought-stressed trees following anaerobic respiration might be prolonged by providing a minimal but adequate amount of water to balance water loss from transpiration.

Along with the type of water stress, a growing body of research indicates that the duration of flooding influences host selection and colonization by ambrosia beetles. Previous studies have indicated that the majority of tunneling by *X. crassiusculus* and *X. germanus* tends to occur during the early to intermediate stages of physiological stress, for instance, within 7–10 days after imposing experimental flood stress (Ranger et al. 19, 26). By flooding container-grown trees for either 3 or 7 days, Reding et al. (18) demonstrated that ambrosia beetle tunnels and the occurrence of offspring tended to increase as flood duration increased. Specifically, the incidence of superficial and healed tunnels was greater in *C. florida* trees flooded for 3 days, while the incidence of deeper tunnels with *X. germanus* foundresses and offspring was greater in *C. florida* trees flooded for 7 days (18). Similarly, more *X. germanus* foundresses and offspring were recovered from *C. florida* and *Malus* *× domestica* trees flooded for 10 days than from trees flooded for 3 and 5 days (18). By simultaneously deploying *C. florida* trees that were flood stressed for varying lengths of time, we subsequently found that more *A. maiche* and *X. germanus* adult foundresses were recovered from trees that were exposed to beetles after 1–16 or 7–22 days of flooding than from trees that were exposed after 14–29 or 28–43 days of flooding. These studies suggest that host tree chemistry plays a crucial role in providing a substrate conducive for ambrosia beetles to cultivate their fungal mutualists and facilitate brood production and fitness. In addition to the presence of ethanol (39, 41, 42), other stress-induced compounds and moisture content likely contribute to mechanisms of host selection, acceptance, and colonization by ambrosia beetles.

A limitation of our current study is that only one tree species (i.e., *C. florida*) was used to test the effect of drought stress on host selection by ambrosia beetles. Future studies could benefit by including other tree species that are drought intolerant, along with drought-tolerant tree species. For instance, when given a choice among trees varying in their tolerance of flood stress, *X. crassiusculus* and *X. germanus* preferentially tunneled into flood-intolerant over flood-tolerant tree species (19). The native habitat of *C. florida* in North America is generally characterized by mesic sites with a moderate amount of moisture (48). *C. florida* is considered flood intolerant due in part to its shallow root system (49, 50). However, *C. florida* is also considered drought intolerant and more intolerant than other deciduous trees, including *Acer saccharum*, *Cornus kousa*, *Cornus racemose*, *Quercus alba*, *Quercus rubra*, and *Quercus velutina* (51, 52).

Global changes in precipitation resulting in water-stress conditions (i.e., drought *vs.* flooding) that predispose trees to infestation by insects represent a significant challenge to producers of horticultural tree crops. Results from our study improve our understanding of how water stress influences the host selection and colonization behavior of exotic ambrosia beetles, thereby contributing to a management strategy. The negative impact of flood stress is greater than drought stress on tree vulnerability to opportunistic ambrosia beetles, which supports Frank and Ranger (24). Duration of flood stress is also of importance, which provides insight into the vulnerability of trees exposed to varying durations of stress. With respect to flood stress, the vulnerability of horticultural tree crops to ambrosia beetles within the midwestern USA is likely to be greatest in April–June due to an increasing frequency of extreme springtime rainfall (53) coinciding with peak flight activity of *X. germanus* and other ambrosia beetles (54, 55). Our current study also contributes to a growing body of evidence supporting the fact that *A. maiche* preferentially infests trees subjected to flood stress, which will aid in developing a management strategy for this recently established exotic ambrosia beetle.

## Data availability statement

The raw data supporting the conclusions of this article will be made available by the authors, without undue reservation.

## Ethics statement

Ethical review and approval was not required for this study on ambrosia beetles in accordance with local legislation and institutional requirements. No protected species were sampled during the course of the experiments.

## Author contributions

CR: resources and project administration, supervision and writing—original draft preparation; CR, MP, SG, JB, SV, JW, FB-G, JO, MR: methodology and analysis, conceptualization, validation and visualization; CR, MP, SG, JB, SV, JW, FB-G, JO, MR: investigation and writing—review and editing. CR, JB, JO, MR: data curation. All authors have read and agreed to the published version of the manuscript.

## References

[1] Hulcr J Stelinski LL. The ambrosia symbiosis: from evolutionary ecology to practical management. Annu Rev Entomol. (2017) 62:285–303. doi: 10.1146/annurev-ento-031616-035105 27860522

[2] Ranger CM Reding ME Addesso K Ginzel M Rassati D. Semiochemical-mediated host selection by *Xylosandrus* spp. ambrosia beetles (Coleoptera: curculionidae) attacking horticultural tree crops: a review of basic and applied science. Can Entomol. (2021) 153:103–20. doi: 10.4039/tce.2020.51

[3] Ranger CM Reding ME Schultz PB Oliver JB Frank SD Addesso KM. Biology, ecology, and management of nonnative ambrosia beetles (Coleoptera: Curculionidae: Scolytinae) in ornamental plant nurseries. J Integ. Pest Manag. (2016) 7(1):1–23. doi: 10.1093/jipm/pmw005

[4] Agnello A Breth D Tee E Cox K Warren HR. Ambrosia beetle–an emergent apple pest. New York Fruit Quart. (2015) 23:25–8. Available at: http://nyshs.org/wp-content/uploads/2015/03/25-28-Agnello-Pages-NYFQ-Book-Spring-2015.eg-5.pdf.

[5] Agnello AM Breth DI Tee EM Cox KD Villani SM Ayer KM. *Xylosandrus germanus* (Coleoptera: curculionidae: scolytinae) occurrence, fungal associations, and management trials in New York apple orchards. J Econ. Entomol. (2017) 110:2149–64. doi: 10.1093/jee/tox189 29048587

[6] Haack RA Rabaglia RJ. Exotic bark and ambrosia beetles in the USA: potential and current invaders. In: Pena J, editor. Potential invasive pests of agricultural crops. Wallingford: CABI Publishing (2013). p. 48–74.

[7] Gomez DF Rabaglia RJ Fairbanks KE Hulcr J. North American Xyleborini north of Mexico: a review and key to genera and species (Coleoptera, Curculionidae, Scolytinae). ZooKeys (2018) 768:19. doi: 10.3897/zookeys.768.24697 PMC6019436 29955211

[8] Rabaglia RJ Cognato AI Hoebeke ER Johnson CW LaBonte JR Carter ME. Early detection and rapid response: a 10-year summary of the USDA Forest Service program of surveillance for non-native bark and ambrosia beetles. Am Entomol. (2019) 65:29–42. doi: 10.1093/ae/tmz015

[9] Rabaglia RJ Vandenberg NJ Acciavatti RE. First records of *Anisandrus maiche* Stark (Coleoptera: Curculionidae: Scolytinae) from North America. Zootaxa (2009) 2137:23–8. doi: 10.11646/zootaxa.2137.1.2

[10] Atkinson TH. Bark and ambrosia beetles of the americas (2022). Available at: http://www.barkbeetles.info (Accessed 13 Dec 2022).

[11] Ranger CM Schultz PB Frank SD Reding ME. Freeze stress of deciduous trees induces attacks by opportunistic ambrosia beetles. Agric For Entomol. (2019) 21:168–79. doi: 10.1111/afe.12317

[12] Tobin KN Ginzel MD. The ambrosia beetle *Anisandrus maiche* (Stark) is repelled by conophthorin and verbenone and attracted to ethanol in a dose-dependent manner. Agric For Entomol. (2023) 25:103–10. doi: 10.1111/afe.12534

[13] Mayers CG Harrington TC Ranger CM. First report of a sexual state in an ambrosia fungus: *Ambrosiella cleistominuta* sp. nov. associated with the ambrosia beetle *Anisandrus maiche* . Botany (2017) 95:503–12. doi: 10.1139/cjb-2016-0297

[14] Harrington TC McNew D Mayers C Fraedrich SW Reed SE. *Ambrosiella roeperi* sp. nov. is the mycangial symbiont of the granulate ambrosia beetle, *Xylosandrus crassiusculus* . Mycologia (2014) 106:835–45. doi: 10.3852/13-354 24895423

[15] Mayers CG McNew DL Harrington TC Roeper RA Fraedrich SW Biedermann PH. Three genera in the ceratocystidaceae are the respective symbionts of three independent lineages of ambrosia beetles with large, complex mycangia. Fungal Biol (2015) 119:1075–92. doi: 10.1016/j.funbio.2015.08.002 26466881

[16] Koricheva J Larsson S Haukioja E. Insect performance on experimentally stressed woody plants: a meta-analysis. Annu Rev Entomol. (1998) 43:195–216. doi: 10.1146/annurev.ento.43.1.195 15012389

[17] Frank SD Anderson AL Ranger CM. Interaction of insecticide and media moisture on ambrosia beetle (Coleoptera: Curculionidae) attacks on selected ornamental trees. Environ Entomol. (2017) 46:1390–96. doi: 10.1093/ee/nvx163 29069311

[18] Reding ME Ranger CM Schultz PB. Colonization of trees by ambrosia beetles (Coleoptera: Curculionidae: Scolytinae) is influenced by duration of flood stress. J Econ. Entomol. (2021) 114:839–47. doi: 10.1093/jee/toab021 33675660

[19] Ranger CM Schultz PB Frank SD Chong JH Reding ME. Non-native ambrosia beetles as opportunistic exploiters of living but weakened trees. PloS One (2015) 10(7):e0131496. doi: 10.1371/journal.pone.0131496 26134522 PMC4489854

[20] Park Williams A Allen CD Macalady AK Griffin D Woodhouse CA Meko DM. Temperature as a potent driver of regional forest drought stress and tree mortality. Nat Climate Change (2013) 3:292–97. doi: 10.1038/nclimate1693

[21] Anderegg WR Hicke JA Fisher RA Allen CD Aukema J Bentz B. Tree mortality from drought, insects, and their interactions in a changing climate. New Phytol (2015) 208:674–83. doi: 10.1111/nph.13477 26058406

[22] Hara AH Beardsley JW. The biology of the black twig borer, *Xylosandrus compactus* (Eichhoff), in Hawaii. Proc Hawaiian Entomol. Soc (1979) 23:55–70. Available at: https://scholarspace.manoa.hawaii.edu/server/api/core/bitstreams/c4dcf8a4-71c4-4111-9ff8-7be235fb9e55/content.

[23] Greco EB Wright MG. Ecology, biology, and management of *Xylosandrus compactus* (Coleoptera: Curculionidae: Scolytinae) with emphasis on coffee in Hawaii. J Integ. Pest Manag. (2015) 6:7. doi: 10.1093/jipm/pmv007

[24] Frank SD Ranger CM. Developing a media moisture threshold for nurseries to reduce tree stress and ambrosia beetle attacks. Environ Entomol. (2016) 45:1040–48. doi: 10.1093/ee/nvw076 27412195

[25] Kelsey RG Joseph G. Attraction of *Scolytus unispinosus* bark beetles to ethanol in water-stressed Douglas-fir branches. For Ecol Manag. (2001) 144:229–38. doi: 10.1016/S0378-1127(00)00387-X

[26] Ranger CM Reding ME Schultz PB Oliver JB. Influence of flood-stress on ambrosia beetle host-selection and implications for their management in a changing climate. Agric For Entomol. (2013) 15:56–64. doi: 10.1111/j.1461-9563.2012.00591.x

[27] Kelsey RG Gallego D Sánchez-García FJ Pajares JA. Ethanol accumulation during severe drought may signal tree vulnerability to detection and attack by bark beetles. Can J For Res (2014) 44:554–61. doi: 10.1139/cjfr-2013-0428

[28] O’Hara RB Kotze DJ. Do not log-transform count data. Methods Ecol Evol (2010) 10:118–22. doi: 10.1111/j.2041-210X.2010.00021.x

[29] Lange S Volkholz J Geiger T Zhao F Vega I Veldkamp T. Projecting exposure to extreme climate impact events across six event categories and three spatial scales. Earth’s Future (2020) 8(12):e2020EF001616. doi: 10.1029/2020EF001616

[30] Pörtner HO Roberts DC Adams H Adler C Aldunce P Ali E. Climate change 2022: impacts, adaptation and vulnerability. Geneva, Switzerland: IPCC (2022). p. 3056.

[31] Devin SR Prudencio ÁS Mahdavi SME Rubio M Martínez-García PJ Martínez-Gómez P. Orchard management and incorporation of biochemical and molecular strategies for improving drought tolerance in fruit tree crops. Plants (2023) 12:773. doi: 10.3390/plants12040773 36840120 PMC9960531

[32] Lindner M Maroschek M Netherer S Kremer A Barbati A Garcia-Gonzalo J. Climate change impacts, adaptive capacity, and vulnerability of European forest ecosystems. For Ecol Manag. (2010) 259:698–709. doi: 10.1016/j.foreco.2009.09.023

[33] Jactel H Petit J Desprez-Loustau ML Delzon S Piou D Battisti A. Drought effects on damage by forest insects and pathogens: a meta-analysis. Global Change Biol (2012) 18:267–76. doi: 10.1111/j.1365-2486.2011.02512.x

[34] Raffa KF Aukema BH Bentz BJ Carroll AL Hicke JA Turner MG. Cross-scale drivers of natural disturbances prone to anthropogenic amplification: the dynamics of bark beetle eruptions. Biosci (2008) 58:501–17. doi: 10.1641/B580607

[35] Bentz BJ Régnière J Fettig CJ Hansen EM Hayes JL Hicke JA. Climate change and bark beetles of the western United States and Canada: direct and indirect effects. BioSci (2010) 60:602–13. doi: 10.1525/bio.2010.60.8.6

[36] Netherer S Panassiti B Pennerstorfer J Matthews B. Acute drought is an important driver of bark beetle infestation in Austrian Norway spruce stands. Front For Glob. Change (2019) 2:39. doi: 10.3389/ffgc.2019.00039

[37] Howe M Peng L Carroll A. Landscape predictions of western balsam bark beetle activity implicate warm temperatures, a longer growing season, and drought in widespread irruptions across British Columbia. For Ecol Manag. (2022) 508:120047. doi: 10.1016/j.foreco.2022.120047

[38] Robbins ZJ Xu C Aukema BH Buotte PC Chitra-Tarak R Fettig CJ. Warming increased bark beetle-induced tree mortality by 30% during an extreme drought in California. Global Change Biol (2022) 28:509–23. doi: 10.1111/gcb.15927 34713535

[39] Cavaletto G Faccoli M Ranger CM Rassati D. Ambrosia beetle response to ethanol concentration and host tree species. J Appl Entomol. (2021) 145:800–09. doi: 10.1111/jen.12895

[40] Manter DK Kelsey RG. Ethanol accumulation in drought-stressed conifer seedlings. Int J Plant Sci (2008) 169:361–69. doi: 10.1086/526462

[41] Ranger CM Biedermann PH Phuntumart V Beligala GU Ghosh S Palmquist DE. Symbiont selection *via* alcohol benefits fungus farming by ambrosia beetles. Proc Natl Acad Sci USA (2018) 115:4447–52. doi: 10.1073/pnas.1716852115 PMC5924889 29632193

[42] Rassati D Contarini M Ranger CM Cavaletto G Rossini L Speranza S. Fungal pathogen and ethanol affect host selection and colonization success in ambrosia beetles. Agric For Entomol. (2020) 22:1–9. doi: 10.1111/afe.12351

[43] Marchin RM Ossola A Leishman MR Ellsworth DS. A simple method for simulating drought effects on plants. Front Plant Sci (2020) 10:1715. doi: 10.3389/fpls.2019.01715 32038685 PMC6985571

[44] Bartlett MK Klein T Jansen S Choat B Sack L. The correlations and sequence of plant stomatal, hydraulic, and wilting responses to drought. (2016). Proc Natl Acad Sci USA (2016) 113:13098–103. doi: 10.1073/pnas.1604088113 PMC5135344 27807136

[45] Trueba S Pan RH Scoffoni C John GP Davis SD Sack L. Thresholds for leaf damage due to dehydration: declines of hydraulic function, stomatal conductance and cellular integrity precede those for photochemistry. New Phytol (2019) 223:134–49. doi: 10.1111/nph.15779 30843202

[46] Poorter H Fiorani F Stitt M Schurr U Finck A Gibon Y. The art of growing plants for experimental purposes: a practical guide for the plant biologist. Funct Plant Biol (2012) 39:821–38. doi: 10.1071/FP12028 32480833

[47] Earl HJ. A precise gravimetric method for simulating drought stress in pot experiments. Crop Sci (2003) 43:1868–73. doi: 10.2135/cropsci2003.1868

[48] Fralish JS Jones SM O'Dell RK Chambers JL. The effect of soil moisture on site productivity and forest composition in the Shawnee hills of southern Illinois. In: Balmer WE, editor. Proceedings of the soil site productivity symposium. Washington, DC: USDA Forest Service (1978). p. 263–85.

[49] Hinckley TM Dougherty PM Lassoie JP Roberts JE Teskey RO. A severe drought: impact on tree growth, phenology, net photosynthetic rate and water relations. Am Midland Nat (1979) 102:307–16. doi: 10.2307/2424658

[50] Day SD Seiler JR Persaud N. A comparison of root growth dynamics of silver maple and flowering dogwood in compacted soil at differing soil water contents. Tree Physiol (2000) 20:257–63. doi: 10.1093/treephys/20.4.257 12651462

[51] Bassuk NL Whitlow T Wittick E. Evaluating street trees for drought tolerance. In: Improving the Quality of Urban Life with Plants, vol. 2. International New York Botanical Garden Symp. on Urban Hort. Publication (1985). p. 174–87.

[52] Kubiske ME Abrams MD Mostoller SA. Stomatal and nonstomatal limitations of photosynthesis in relation to the drought and shade tolerance of tree species in open and understory environments. Trees (1996) 11:76–82. doi: 10.1007/s004680050062

[53] Feng Z Leung LR Hagos S Houze RA Burleyson CD Balaguru K. More frequent intense and long-lived storms dominate the springtime trend in central US rainfall. Nat Comm. (2016) 7:13429. doi: 10.1038/ncomms13429 PMC5114602 27834368

[54] Oliver JB Mannion CM. Ambrosia beetle (Coleoptera: Scolytidae) species attacking chestnut and captured in ethanol-baited traps in middle Tennessee. Environ Entomol. (2001) 30:909–18. doi: 10.1603/0046-225X-30.5.909

[55] Reding M Oliver J Schultz P Ranger C. Monitoring flight activity of ambrosia beetles in ornamental nurseries with ethanol-baited traps: influence of trap height on captures. J Environ Hort. (2010) 28:85–90. doi: 10.24266/0738-2898-28.2.85

