# Supplementary material for: Type and duration of water stress influence host selection and colonization by exotic ambrosia beetles (Coleoptera: Curculionidae)
Source: Front Insect Sci. 2023 Jul 7;3:1219951. doi: 10.3389/finsc.2023.1219951 (PMC10926373; doi:10.3389/finsc.2023.1219951)
Supplement: Supplementary file 1 [file Table_1.pdf]

Table S1. Output from statistical analyses comparing the number of *Xylosandrus germanus* ambrosia beetles that were entrapped in Tanglefoot applied to the stems of flood stressed, drought stressed, and standard irrigation *Cornus florida* trees (See Fig. 2).

Day	χ^2	df	*P*
1	0.47	2	0.79
4	13.05	2	0.002
6	17.27	2	0.0002
8	19.13	2	<0.0001
10	19.20	2	<0.0001
12	18.18	2	0.0001
14	25.81	2	<0.0001
