# Supplementary material for: Type and duration of water stress influence host selection and colonization by exotic ambrosia beetles (Coleoptera: Curculionidae)
Source: Front Insect Sci. 2023 Jul 7;3:1219951. doi: 10.3389/finsc.2023.1219951 (PMC10926373; doi:10.3389/finsc.2023.1219951)
Supplement: Supplementary file 2 [file Table_2.pdf]

Table S2. Output from statistical analyses comparing the number of ambrosia beetle attacks on flood stressed, drought stressed, and standard irrigation *Cornus florida* trees (See Fig. 3A).

Day	χ^2	df	*P*
1	0.00	2	1.0
2	1.28	2	0.53
3	10.97	2	0.004
4	16.63	2	0.0002
7	27.30	2	<0.0001
8	28.60	2	<0.0001
9	30.06	2	<0.0001
11	30.09	2	<0.0001
14	31.26	2	<0.0001
