# Supplementary material for: Type and duration of water stress influence host selection and colonization by exotic ambrosia beetles (Coleoptera: Curculionidae)
Source: Front Insect Sci. 2023 Jul 7;3:1219951. doi: 10.3389/finsc.2023.1219951 (PMC10926373; doi:10.3389/finsc.2023.1219951)
Supplement: Supplementary file 3 [file Table_3.pdf]

Table S3. Output from statistical analyses comparing the number of ambrosia beetle attacks on flood stressed, drought stressed, and standard irrigation *Cornus florida* trees (See Fig. 3B).

Day	χ^2	df	*P*
1	0.00	2	1.0
2	0.00	2	1.0
3	16.40	2	0.0003
5	43.33	2	<0.0001
8	48.19	2	<0.0001
11	58.13	2	<0.0001
15	57.81	2	<0.0001
19	58.08	2	<0.0001
22	59.07	2	<0.0001
