# Supplementary material for: Type and duration of water stress influence host selection and colonization by exotic ambrosia beetles (Coleoptera: Curculionidae)
Source: Front Insect Sci. 2023 Jul 7;3:1219951. doi: 10.3389/finsc.2023.1219951 (PMC10926373; doi:10.3389/finsc.2023.1219951)
Supplement: Supplementary file 4 [file Table_4.pdf]

Table S4. Output from statistical analyses comparing the number of ambrosia beetle attacks on *Cornus florida* trees subjected to varying durations of flooding (see Fig. 4).

Day	χ^2	df	*P*
1	25.36	4	<0.0001
3	47.53	4	<0.0001
6	59.26	4	<0.0001
8	64.14	4	<0.0001
10	66.61	4	<0.0001
13	67.24	4	<0.0001
15	67.38	4	<0.0001
